# A reaction-diffusion model of cholinergic retinal waves and self-organized criticality

**DOI:** 10.1186/1471-2202-14-S1-P230

**Published:** 2013-07-08

**Authors:** Benjamin J Lansdell, J Nathan Kutz

**Affiliations:** 1 Department of Applied Mathematics, University of Washington, Seattle, WA 98195, USA

## 

**Figure 1:**
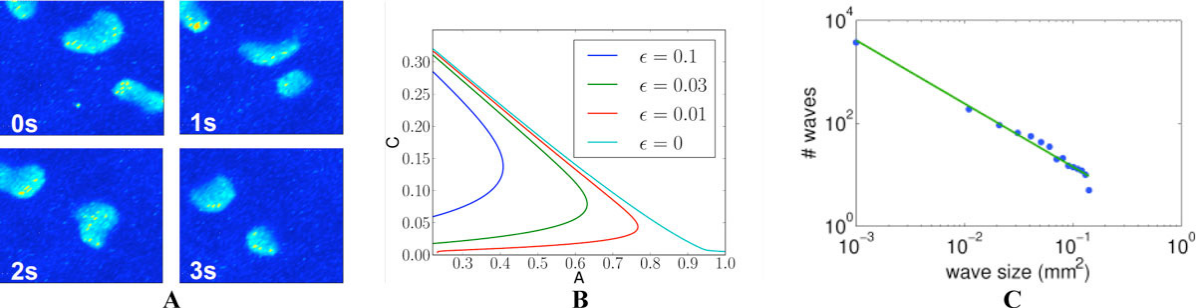
A: Simulated voltage dynamics of model. SACs have Morris-Lecar dynamics, with local coupling occurring via diffusion of extracellular ACh, and a noisy channel to induce spontaneous firing. B: Traveling wave speed *C *as a function of threshold parameter *A *and refractory timescale *ε*, computed through numerical continuation. C: Blue points indicate frequency of wave-sizes from 5000s of simulation. Green line indicates fitted power-law distribution with exponent -1.23, coefficient of determination r^2 ^= 0.98.

Prior to receiving visual stimuli, spontaneous, correlated activity called retinal waves drives activity-dependent developmental programs. Early-stage waves mediated by acetylcholine (ACh) manifest as slow, spreading bursts of action potentials. They are believed to be initiated by the spontaneous firing of Starburst Amacrine Cells (SACs), whose dense, recurrent connectivity then propagates this activity laterally. Their extended inter-wave intervals and shifting wave boundaries are the result of the slow after-hyperpolarization of the SACs creating an evolving mosaic of recruitable and refractory cells, which can and cannot participate in waves. Recent evidence suggests that cholinergic waves may be modulated by the extracellular concentration of ACh [1].Here, we have constructed a simplified, yet biophysically realistic, reaction-diffusion model of cholinergic retinal waves capable of recapitulating wave dynamics observed in mice retina recordings (Figure 1A). The dense, recurrent connectivity of SACs is modeled through local, excitatory coupling occurring via the volume release and diffusion of ACh. This novel approach is used to determine how extracellular ACh may modulate wave activity. In contrast with previous, simulation-based models (*e.g*. the model of Hennig [2]), we are able to use non-linear wave theory (traveling fronts, pulses, singular perturbation analysis, *etc*.) to connect wave features to underlying physiological parameters, making our model useful in determining appropriate pharmacological manipulations to experimentally produce waves of a prescribed spatiotemporal character (Figure 1B).

This is the first mathematical analysis of its type on retinal waves. However, a number of theoretical issues remain unresolved. The distribution of wave sizes has been reported to obey a power-law distribution, suggesting the developing retina may exist in a critical state [2]. Are these findings compatible with our theoretical model? We present preliminary results suggesting that our model possesses a configuration in which wave sizes are distributed according to a power-law (Figure 1C). We adapt analyses typically used in neural field equations to understand the effects of stochasticity and heterogeneity on wave size statistics [3], and therefore provide theoretical arguments characterizing the potential for criticality in retinal development.
